# Mechanical Dentistry as an Art—Its Decline

**Published:** 1872-01

**Authors:** H. A. Smith


### MECHANICAL DENTISTRY—ITS DECLINE AS AN ART.

BY DR. H. A. SMITH.

Read before the Ohio State Society December 6, 1871.

Any one, who has observed with care the proceedings of Dental Associations for the past ten or fifteen years, could not fail to have noticed a gradual decline of interest, on the part of the profession, in the department of mechanical dentistry.

In our own society, since its organization in 1866, camparatively little time has been given to the consideration of this subject. And it is to be regretted that what has been said was not always of a character to aid in elevating this specialty to its proper place as an art.

When questioned as to the cause of this state of things, it is usual to attribute it to the introduction and general use of vulcanite and other cheap bases for artificial teeth.

The use of the inferior bases has done very much in fixing the estimate which both the public and profession have of mechanical dentistry. Yet there is another cause which, in my judgment, underlies this—a cause that has had the effect to lower the standard of art in other departments than this specialty of ours. I refer to a *marked tendency of the age to render art mechanical.*

In *painting* it takes form in attempting to re-produce by mechanical means, as in the chromo-lithograph, the "subtle delicacies of color in good pictorial art," which can be alone produced by the dexterous hand of the trained artist.

In *architecture* it is observed in the frequent reproduction of styles or forms, especially those which may be produced by machinery, giving to the streets of our cities an effect which is cold and formal; also, in the cheap and insecure manner of building, so that it can no longer be said a man's house is his castle.

In our *furniture* the same tendency is too often observed, by sacrificing the true design and usefulness of the article to a gaudy, perishable style, profusely decorated with machine carvings.

This tendency to render art mechanical became apparent in mechanical dentistry *when artificial teeth were first successfully molded.*

It is usual to assert, and it is generally believed, that the period when carved teeth were abandoned and mechanically-made teeth used in their stead, marked an era of great advancement in mechanical dentistry. On the contrary, I think it may be shown that this was the starting point in the decline of true artistic mechanical dentistry.

This is not so apparent to us now, as it would have been had the art of carving teeth been practiced at a period when the tendency we have named—to re produce, cheaply and rapidly, all classes of art work—had not prevailed. As it is, the art of carving teeth may properly be said to have become a *lost art* before it was yet fully developed. Most of us, however, have seen specimens of such teeth that gave evidence of a talent in this direction, which, had it been encouraged and developed, would have produced a race of carvers of artificial teeth who would have taken a high rank as true artists. Skilled in the use of the hands, they also would have exercised the intelligence and emotions, the use of which are essential to rise above the mere mechanical operator.

Being successful in manufacturing single teeth, it naturally followed that blocks of two or more should be fashioned in the same way. When this was done the decline of the art was still more noticeable.

In the use of single teeth, with gum, good results were produced, in the hands of the trained dentist. Their use gave him some freedom in the arrangement, and every case from his hands had the merit of his own individuality. When single teeth without gum were used, the gum being afterwards supplied, still more satisfactory and natural artificial teeth were made. Although the dentist may have sacrificed somewhat his conceptions of the appearance of the case, in using teeth shaped by another, he still had left sufficient latitude to exercise the artistic spirit.

Now, let a man, trained in such a school of artificial dentistry, be compelled to use teeth manufactured in blocks, will he not find himself fettered? Having been arranged and fashioned by another, he can not change the character and expression—if, indeed, they possess such qualities—neither can he adapt the teeth, one to the other, as he would like. Being fixed and rigid in arrangement, they must be placed as designed in the beginning. The most he can do is to arrange the several blocks with close fitting joints and smooth gum. If the case, when placed in the mouth, has any of the elements of an artistic set of teeth, the dentist, if he is a conscientious man, will be compelled to give a large share of the praise to the designer and molder of the blocks which he has used?

Has it never occurred to you, when selecting teeth from the great variety before you, that whatever of the artistic element there is left in mechanical dentistry is due largely to the designer and molder of the teeth? The dentist, who exercises his intelligence and skill in selecting a set of artificial teeth for his case, may be an artist, in some degree; but is not he who has shaped, arranged and colored them for his use, entitled to the same consideration? Yet the occupation of the former we would regard as professional, and the latter that only of a skilled workman.

However we may differ as to the extent of the influence the cause we have named has had in lowering the standard of mechanical dentistry, both in the estimation of the public and the profession, all will agree that the introduction and use of vulcanite and other cheap bases has had a tendency to rapidly bring the art down to nearly the level of a trade.

It may be stated as an *axiom* in the practice of dentistry, as well as in the arts generally, that the use of an inferior material induces inferior workmanship. The artist does not expend his time and talents upon a subject wrought in a perishable material. In order to call forth his best efforts, he must have constantly before him the idea that his work will be lasting.

Ruskin, in one of his works on art, relates that tradition tells us that Michael Angelo was once commanded to mold a statue out of snow, by Pietro di Medicii, and that he, in obedience, executed a beautiful work of art. Michael Angelo's fame still lives, but it is due to the fact that he did not habitually spend his time and talents upon a material that would perish with the seasons. He wrought in the enduring marble, and his reputation is as lasting as time.

The use of the precious metals in mechanical dentistry gives us the assurance of great durability, and no expenditure of time and care is thought to be wasted. The thought that a set of artificial teeth cannot be of any permanent value necessarily induces a hurried and careless habit of manipulation and a consequent lowering of the art. Besides, there is something in the working of these metals—their solidity and power to resist chemical change—their value—that cultivates steadiness of hand and caution. Hence we often see work of this kind that seems to have been made with the dentist's whole heart and soul in it.

It was formerly thought that the training which the use of the precious metals in mechanical dentistry gives the student was admirably adapted to fit him for operative dentistry; and it is true that it teaches him the precision and firmness of hand so essential to success in this department. On the other hand, the working of vulcanite aud other cheap bases induces a hurried, loose and infirm style of manipulation that is necessarily fatal to good results in operations upon the natural teeth.

Inferior workmanship implies inferior workmen. Hence we find the mechanical department crowded with dentists who have come into the profession without either the cost of time or money. They are too often men who are a reproach to the calling, since their only ambition appears to be to manufacture the greatest number of sets of artificial teeth with the least expenditure of time or thought. In the commercial world the public appear to discriminate in most occupations between the skilled and the unskilled. The brick-layer is paid more than the man who mixes his mortar. But in mechanical dentistry the tendency is to force the competent dentist down to a level with the inferior man, by placing him directly in competition with the latter. The result has been that the field is almost entirely abandoned to those who have no conception of what belongs to a true art calling. Those of the profession who have the training and the ability to develop the true artistic spirit of mechanical dentistry have had their interest enlisted in other departments of dentistry, and, very naturally, they have ceased to talk upon this subject, as was once the custom.

